# Supplementary figures and images for: Genomic analysis of the tryptome reveals molecular mechanisms of gland cell evolution
Source: EvoDevo. 2019 Sep 30;10:23. doi: 10.1186/s13227-019-0138-1 (PMC6767649; doi:10.1186/s13227-019-0138-1)

## Slide 1
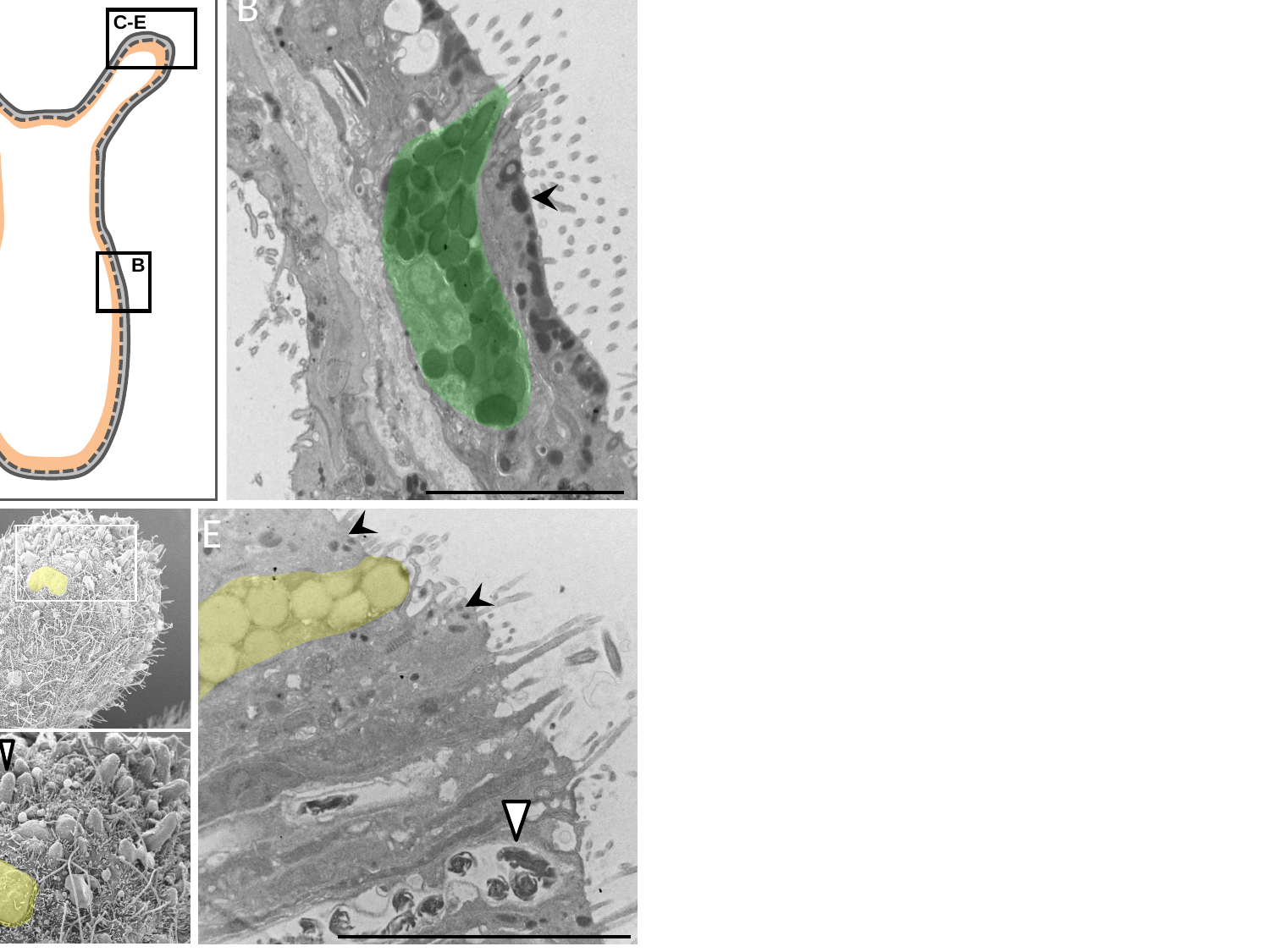

Additional file 1
A
B
C-E
B
E
C
D

Supplement: Supplementary file 1 — Additional file 1. Gland cells of the external ectoderm. a–c Mucus cells (false colored yellow) in the tentacle ectoderm. d Zymogen cell (false colored green) in the body wall ectoderm. White arrows point to cnidocytes, black arrowheads point to electron dense apical vesicles in cells adjacent to gland cells. a, b SEM, c, d TEM. Scale bars: black—5 µm, white—10 µm. [file 13227_2019_138_MOESM1_ESM.pptx]

## Slide 1
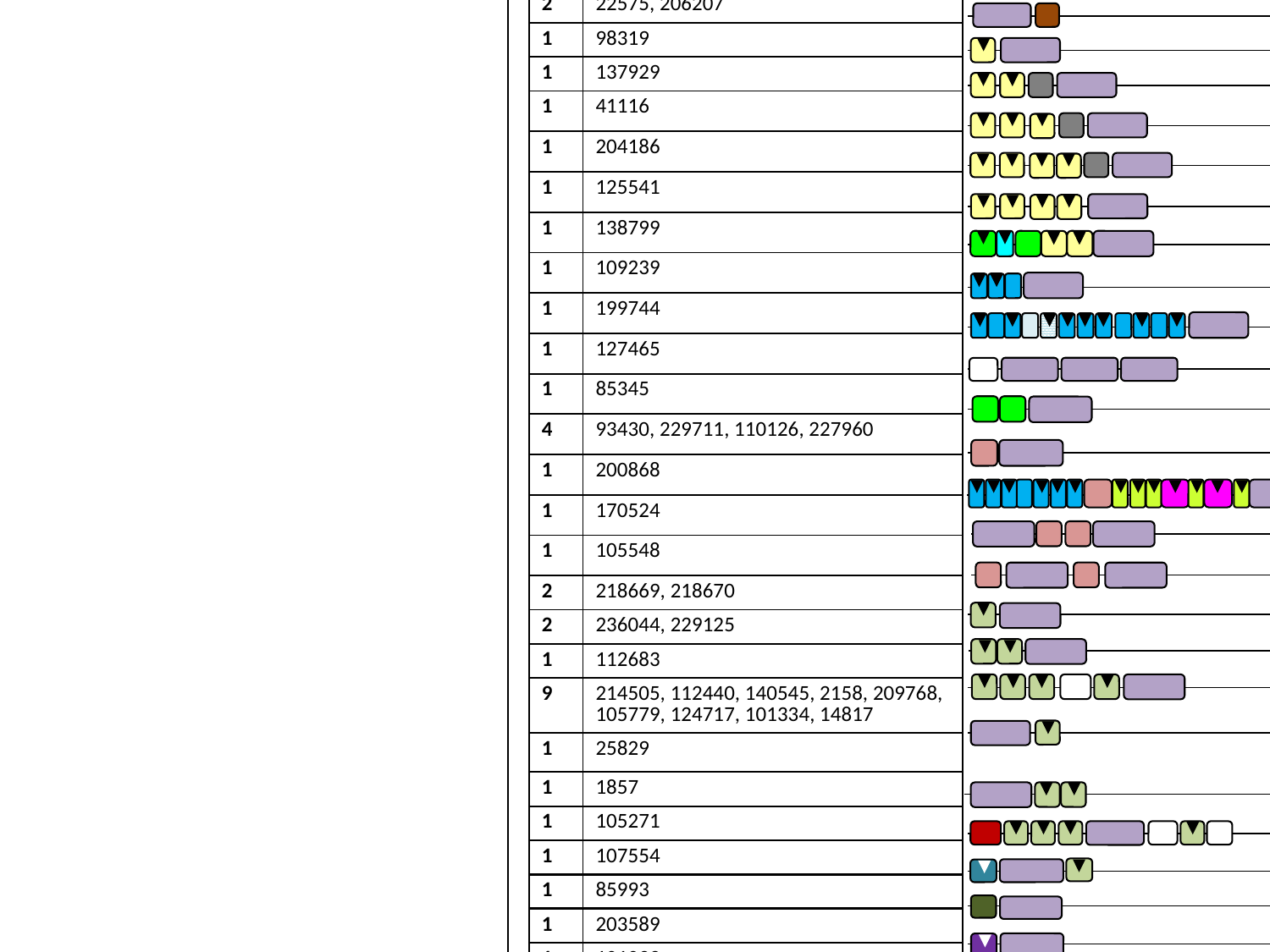

Additional file 5
NVJ Protein ID
Single-exon domain
| 26 | … |
| --- | --- |
| 2 | 128003, 216003 |
| 2 | 22575, 206207 |
| 1 | 98319 |
| 1 | 137929 |
| 1 | 41116 |
| 1 | 204186 |
| 1 | 125541 |
| 1 | 138799 |
| 1 | 109239 |
| 1 | 199744 |
| 1 | 127465 |
| 1 | 85345 |
| 4 | 93430, 229711, 110126, 227960 |
| 1 | 200868 |
| 1 | 170524 |
| 1 | 105548 |
| 2 | 218669, 218670 |
| 2 | 236044, 229125 |
| 1 | 112683 |
| 9 | 214505, 112440, 140545, 2158, 209768, 105779, 124717, 101334, 14817 |
| 1 | 25829 |
| 1 | 1857 |
| 1 | 105271 |
| 1 | 107554 |
| 1 | 85993 |
| 1 | 203589 |
| 1 | 101093 |
| 1 | 164017 |
| 1 | 109826 |
| 1 | 163196 |
| 1 | 199428 |
+

Supplement: Supplementary file 5 — Additional file 5. All trypsin domains are encoded by multiple exons in N. vectensis (excluding the NVJ_128003 and NVJ_216003) but many of the associated domains are encoded by a single exon (indicated by triangle). Domains that span intron/exon boundaries by ten or fewer nucleotides were considered to be encoded by a single exon. [file 13227_2019_138_MOESM5_ESM.pptx]
