# Supplementary material for: Genomic analysis of the tryptome reveals molecular mechanisms of gland cell evolution
Source: EvoDevo. 2019 Sep 30;10:23. doi: 10.1186/s13227-019-0138-1 (PMC6767649; doi:10.1186/s13227-019-0138-1)
Supplement: Supplementary file 2 — Additional file 2. Phylogeny of Trypsin_2 domains across animals. Protein models for N. vectensis are shown. NVJ_203589 and NVJ_23745 were not detected by the trypsin HMM and do not appear in Fig. 5. Colors as in Fig. 4a. [file 13227_2019_138_MOESM2_ESM.pptx]

## Slide 1
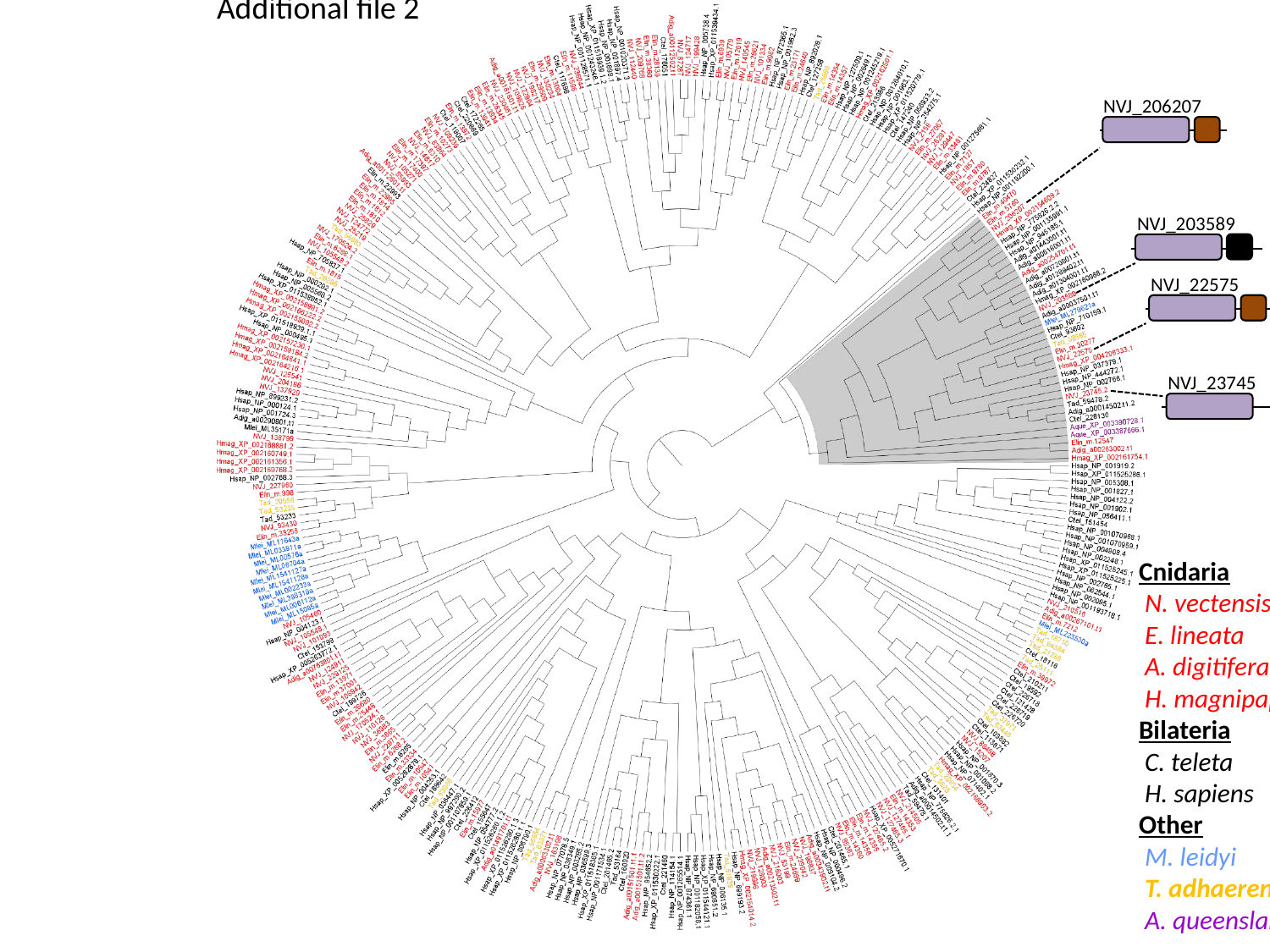

Additional file 2
NVJ_206207
NVJ_203589
NVJ_22575
NVJ_23745
Cnidaria
 N. vectensis
 E. lineata
 A. digitifera
 H. magnipapillata
Bilateria
 C. teleta
 H. sapiens
Other
 M. leidyi
 T. adhaerens
 A. queenslandica
