# Supplementary material for: Genomic analysis of the tryptome reveals molecular mechanisms of gland cell evolution
Source: EvoDevo. 2019 Sep 30;10:23. doi: 10.1186/s13227-019-0138-1 (PMC6767649; doi:10.1186/s13227-019-0138-1)
Supplement: Supplementary file 3 — Additional file 3. Human trypsin domain architecture mapped on the animal trypsin phylogeny. Proteins with multiple trypsin domains are polyphyletic; in such cases, the diagram points to a single trypsin domain and the position of the other trypsin domains is indicated by symbols. N. vectensis proteins characterized by in situ hybridization are indicated by arrows (this study), †[34], or *[12]. [file 13227_2019_138_MOESM3_ESM.pptx]

## Slide 1
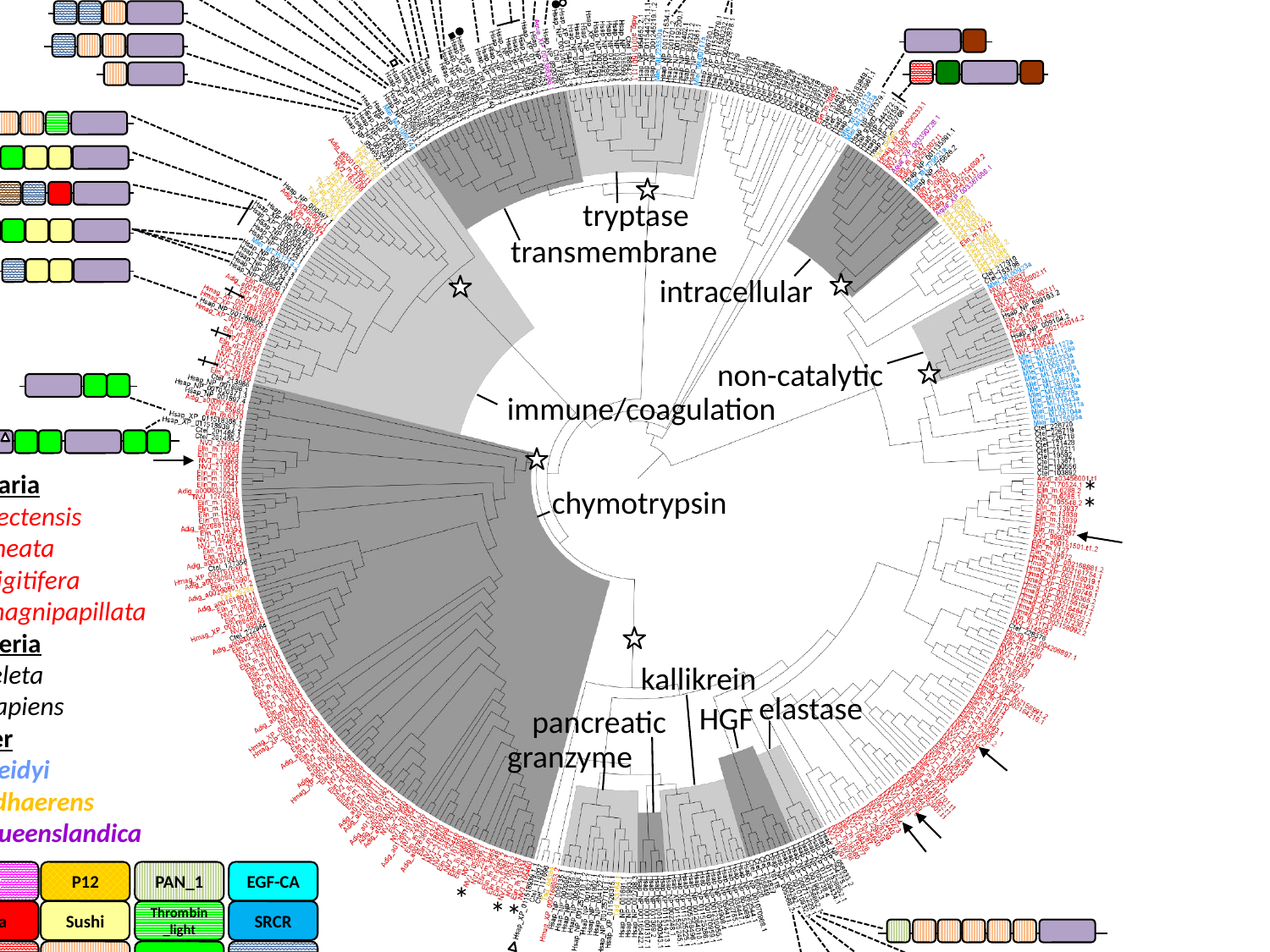

Additional file 3
tryptase
transmembrane
intracellular
†
†
†
non-catalytic
immune/coagulation
Cnidaria
 N. vectensis
 E. lineata
 A. digitifera
 H. magnipapillata
Bilateria
 C. teleta
 H. sapiens
Other
 M. leidyi
 T. adhaerens
 A. queenslandica
*
chymotrypsin
*
kallikrein
elastase
HGF
pancreatic
granzyme
Fz
P12
PAN_1
EGF-CA
*
*
*
FXa
Sushi
Thrombin_light
SRCR
IGFBP
Kringle
CUB
EGF
GVQW
MAM
Kazal_1
SEA
PDZ
Trypsin
fn2
VWA
Gla
V-set
Ldl_a
fn1
