# Supplementary material for: Genomic analysis of the tryptome reveals molecular mechanisms of gland cell evolution
Source: EvoDevo. 2019 Sep 30;10:23. doi: 10.1186/s13227-019-0138-1 (PMC6767649; doi:10.1186/s13227-019-0138-1)
Supplement: Supplementary file 6 — Additional file 6. Trypsin protein domain architectures from all taxa examined in this study. [file 13227_2019_138_MOESM6_ESM.pptx]

## Slide 1
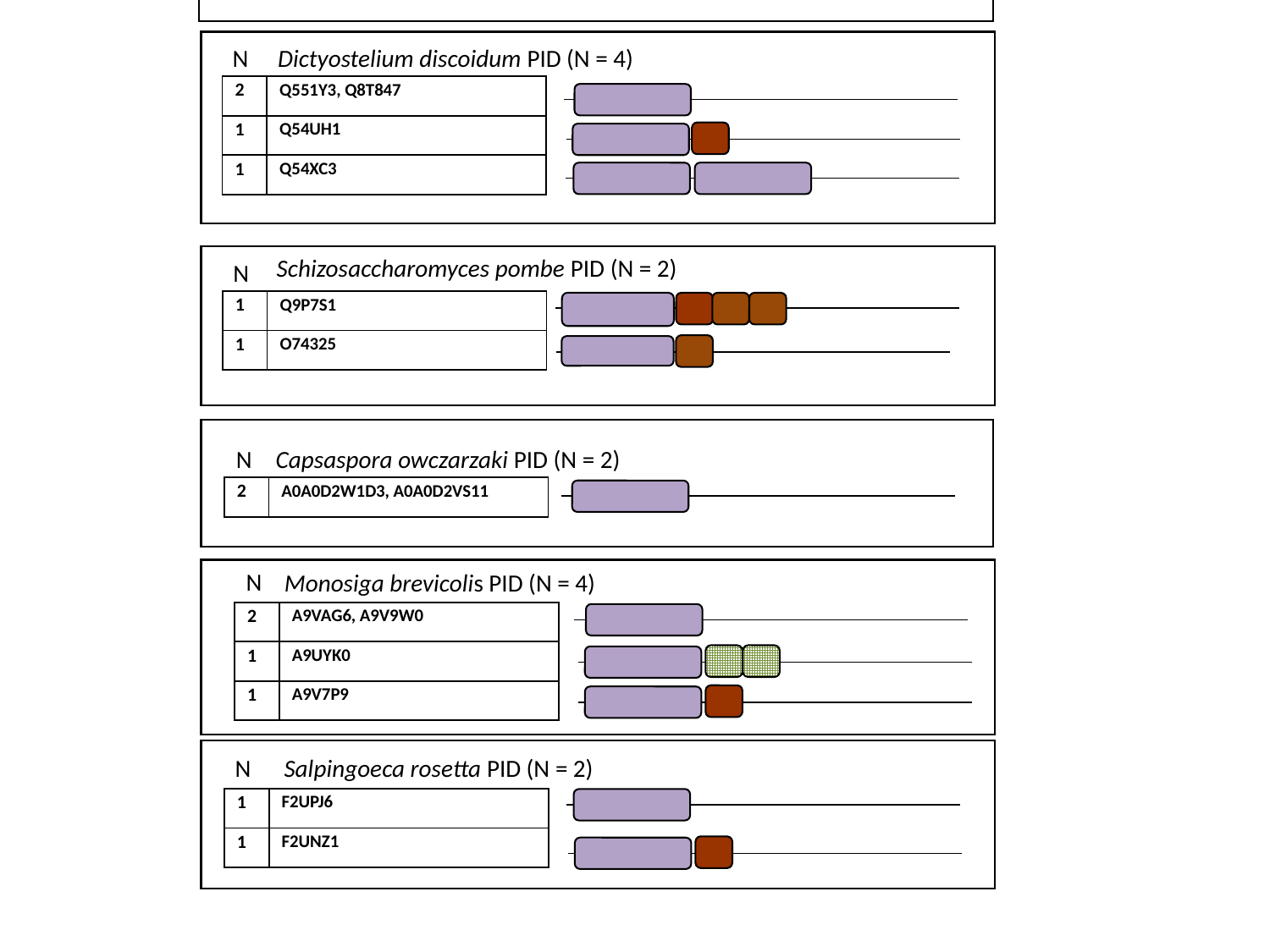

Additional file 6
m.21993
N
Non_eukaryotes PID (N = 4)
m.21993
Trypsin
| 1 | EAW42019 |
| --- | --- |
| 2 | EAW40153, EAW40225 |
| 1 | EAW41081 |
PDZ
Porin_3
m.21993
N
Dictyostelium discoidum PID (N = 4)
| 2 | Q551Y3, Q8T847 |
| --- | --- |
| 1 | Q54UH1 |
| 1 | Q54XC3 |
Schizosaccharomyces pombe PID (N = 2)
m.21993
N
| 1 | Q9P7S1 |
| --- | --- |
| 1 | O74325 |
m.21993
N
Capsaspora owczarzaki PID (N = 2)
| 2 | A0A0D2W1D3, A0A0D2VS11 |
| --- | --- |
m.21993
N
Monosiga brevicolis PID (N = 4)
| 2 | A9VAG6, A9V9W0 |
| --- | --- |
| 1 | A9UYK0 |
| 1 | A9V7P9 |
m.21993
N
Salpingoeca rosetta PID (N = 2)
| 1 | F2UPJ6 |
| --- | --- |
| 1 | F2UNZ1 |

## Slide 2
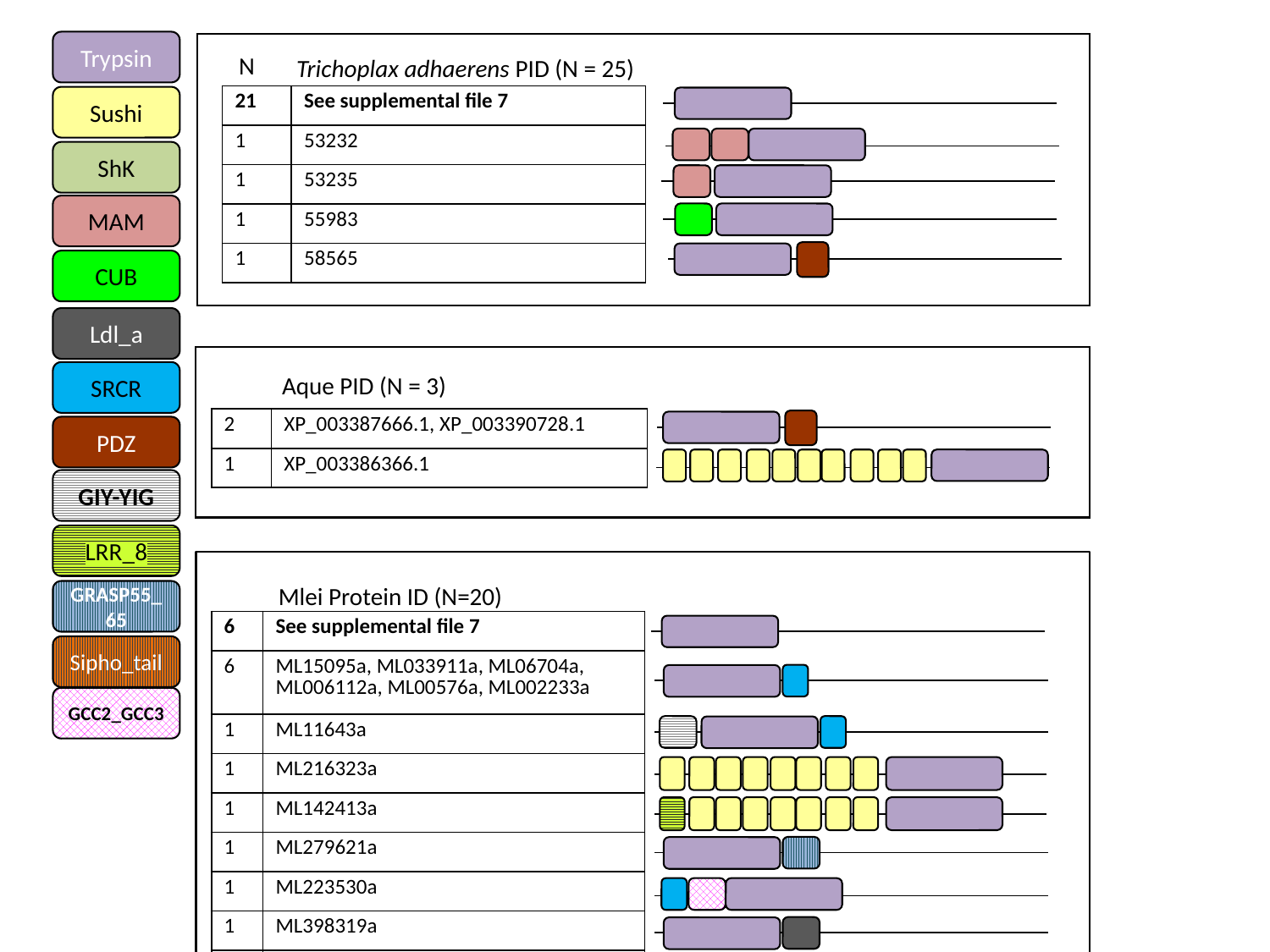

m.21993
Trypsin
m.21993
N
Trichoplax adhaerens PID (N = 25)
| 21 | See supplemental file 7 |
| --- | --- |
| 1 | 53232 |
| 1 | 53235 |
| 1 | 55983 |
| 1 | 58565 |
Sushi
ShK
MAM
CUB
Ldl_a
SRCR
Aque PID (N = 3)
N
| 2 | XP\_003387666.1, XP\_003390728.1 |
| --- | --- |
| 1 | XP\_003386366.1 |
PDZ
GIY-YIG
LRR_8
Mlei Protein ID (N=20)
GRASP55_
65
| 6 | See supplemental file 7 |
| --- | --- |
| 6 | ML15095a, ML033911a, ML06704a, ML006112a, ML00576a, ML002233a |
| 1 | ML11643a |
| 1 | ML216323a |
| 1 | ML142413a |
| 1 | ML279621a |
| 1 | ML223530a |
| 1 | ML398319a |
| 1 | ML00923a |
| 1 | ML00717a |
Sipho_tail
N
GCC2_GCC3

## Slide 3
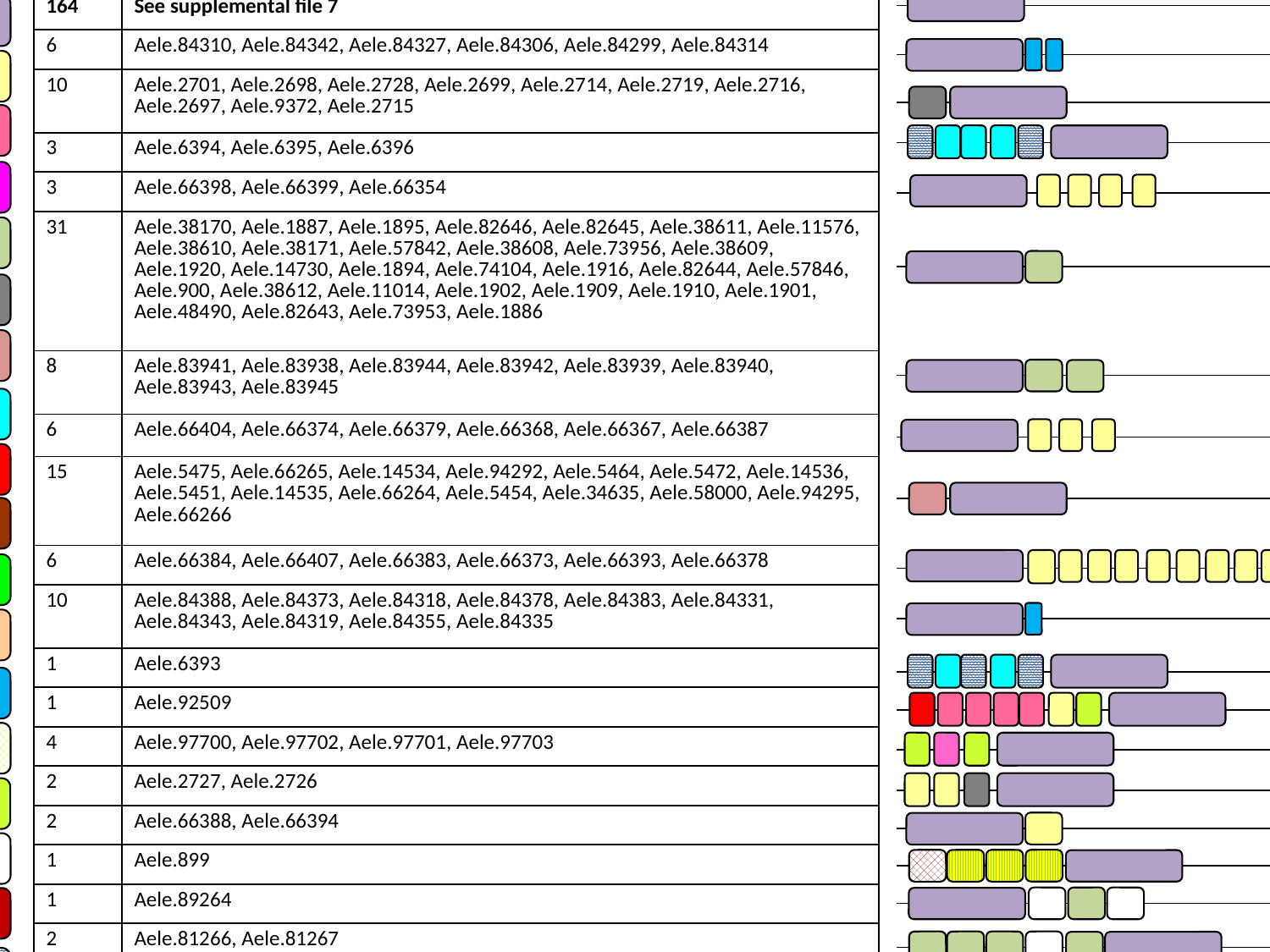

m.21993
N
Anthopleura elegantissima PID (N = 323)
| 164 | See supplemental file 7 |
| --- | --- |
| 6 | Aele.84310, Aele.84342, Aele.84327, Aele.84306, Aele.84299, Aele.84314 |
| 10 | Aele.2701, Aele.2698, Aele.2728, Aele.2699, Aele.2714, Aele.2719, Aele.2716, Aele.2697, Aele.9372, Aele.2715 |
| 3 | Aele.6394, Aele.6395, Aele.6396 |
| 3 | Aele.66398, Aele.66399, Aele.66354 |
| 31 | Aele.38170, Aele.1887, Aele.1895, Aele.82646, Aele.82645, Aele.38611, Aele.11576, Aele.38610, Aele.38171, Aele.57842, Aele.38608, Aele.73956, Aele.38609, Aele.1920, Aele.14730, Aele.1894, Aele.74104, Aele.1916, Aele.82644, Aele.57846, Aele.900, Aele.38612, Aele.11014, Aele.1902, Aele.1909, Aele.1910, Aele.1901, Aele.48490, Aele.82643, Aele.73953, Aele.1886 |
| 8 | Aele.83941, Aele.83938, Aele.83944, Aele.83942, Aele.83939, Aele.83940, Aele.83943, Aele.83945 |
| 6 | Aele.66404, Aele.66374, Aele.66379, Aele.66368, Aele.66367, Aele.66387 |
| 15 | Aele.5475, Aele.66265, Aele.14534, Aele.94292, Aele.5464, Aele.5472, Aele.14536, Aele.5451, Aele.14535, Aele.66264, Aele.5454, Aele.34635, Aele.58000, Aele.94295, Aele.66266 |
| 6 | Aele.66384, Aele.66407, Aele.66383, Aele.66373, Aele.66393, Aele.66378 |
| 10 | Aele.84388, Aele.84373, Aele.84318, Aele.84378, Aele.84383, Aele.84331, Aele.84343, Aele.84319, Aele.84355, Aele.84335 |
| 1 | Aele.6393 |
| 1 | Aele.92509 |
| 4 | Aele.97700, Aele.97702, Aele.97701, Aele.97703 |
| 2 | Aele.2727, Aele.2726 |
| 2 | Aele.66388, Aele.66394 |
| 1 | Aele.899 |
| 1 | Aele.89264 |
| 2 | Aele.81266, Aele.81267 |
| 2 | Aele.66258, Aele.66259 |
| 2 | Aele.53314, Aele.53315 |
| 2 | Aele.2720, Aele.2723 |
| 1 | Aele.25222 |
| 1 | Aele.77727 |
| 3 | Aele.48487, Aele.48486, Aele.48488 |
| 3 | Aele.17373, Aele.17374, Aele.17377 |
| 2 | Aele.62285, Aele.62282 |
| 1 | Aele.45455 |
| 1 | Aele.61797 |
| 1 | Aele.6389 |
| 2 | Aele.82630, Aele.82631 |
| 2 | Aele.89263, Aele.89262 |
| 2 | Aele.76119, Aele.76122 |
| 1 | Aele.13824 |
| 1 | Aele.56925 |
| 1 | Aele.89888 |
| 1 | Aele.89890 |
| 11 | Aele.39884, Aele.39813, Aele.39820, Aele.39774, Aele.39897, Aele.39727, Aele.39871, Aele.39821, Aele.39864, Aele.39850, Aele.39922 |
| 1 | Aele.25224 |
| 1 | Aele.34630 |
| 3 | Aele.76121, Aele.76118, Aele.76120 |
| 1 | Aele.6392 |
| 1 | Aele.92111 |
Trypsin
Sushi
Ldl_b
I-set
ShK
VWA
MAM
EGF-CA
FXa
PDZ
CUB
Thy_1
SRCR
Gly_rich
Ig_2
F5_F8
Astacin
EGF
TSP_1
Ldl_a
WAP
PLAT
fn2
Laminin_N
Laminin_EGF

## Slide 4
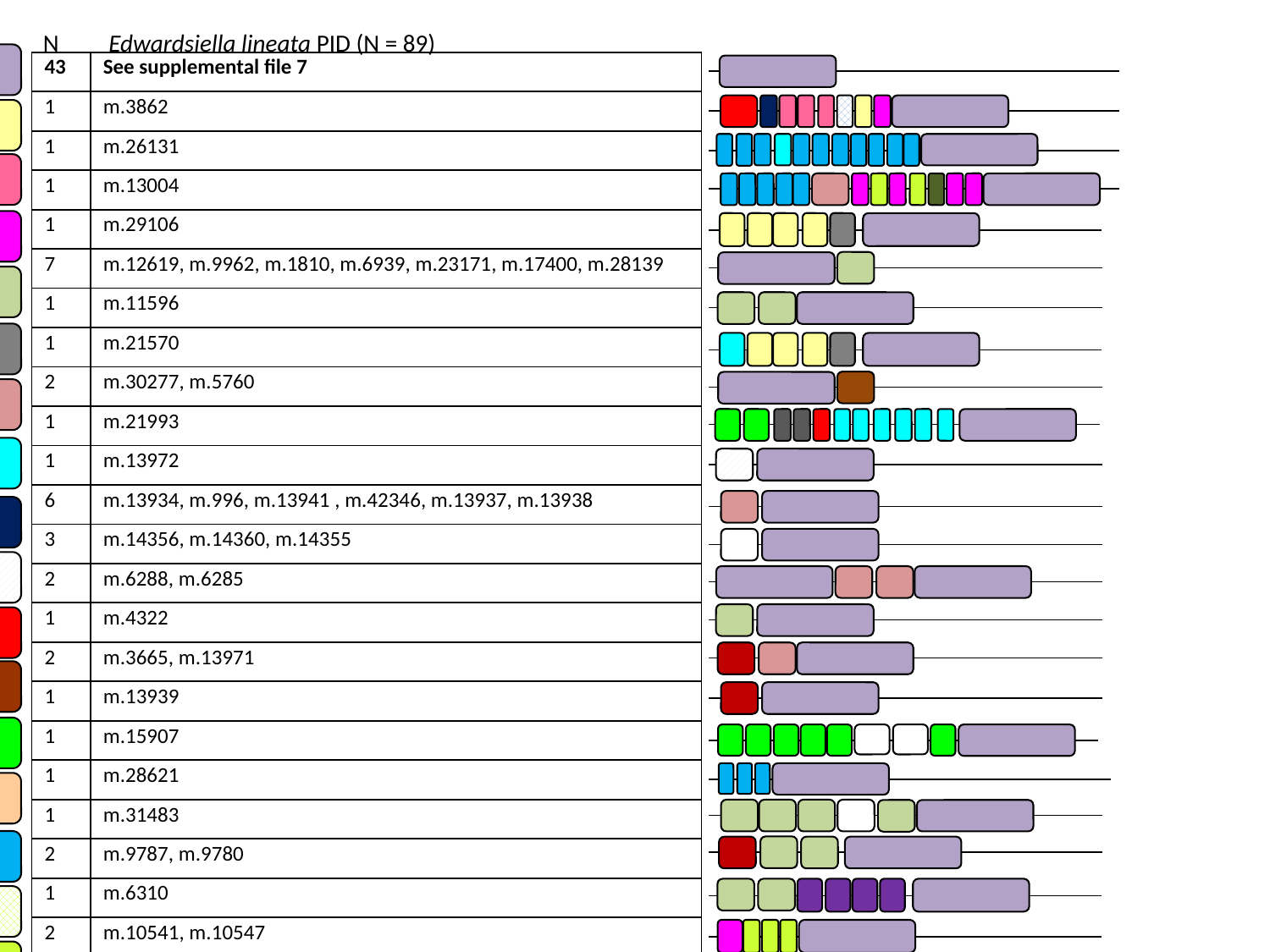

m.21993
Edwardsiella lineata PID (N = 89)
N
Trypsin
| 43 | See supplemental file 7 |
| --- | --- |
| 1 | m.3862 |
| 1 | m.26131 |
| 1 | m.13004 |
| 1 | m.29106 |
| 7 | m.12619, m.9962, m.1810, m.6939, m.23171, m.17400, m.28139 |
| 1 | m.11596 |
| 1 | m.21570 |
| 2 | m.30277, m.5760 |
| 1 | m.21993 |
| 1 | m.13972 |
| 6 | m.13934, m.996, m.13941 , m.42346, m.13937, m.13938 |
| 3 | m.14356, m.14360, m.14355 |
| 2 | m.6288, m.6285 |
| 1 | m.4322 |
| 2 | m.3665, m.13971 |
| 1 | m.13939 |
| 1 | m.15907 |
| 1 | m.28621 |
| 1 | m.31483 |
| 2 | m.9787, m.9780 |
| 1 | m.6310 |
| 2 | m.10541, m.10547 |
| 1 | m.6241 |
| 1 | m.31802 |
| 1 | m.41352 |
| 1 | m.10532 |
| 1 | m.20616 |
| 1 | m.13584 |
Sushi
Ldl_b
I-set
ShK
VWA
MAM
EGF-CA
Lustrin_
cystein
Lectin_C
FXa
PDZ
CUB
Thy_1
SRCR
Gly_rich
Ig_2
F5_F8
Astacin
ETS-PEA3_N
TSP_1
Ldl_a
SGL

## Slide 5
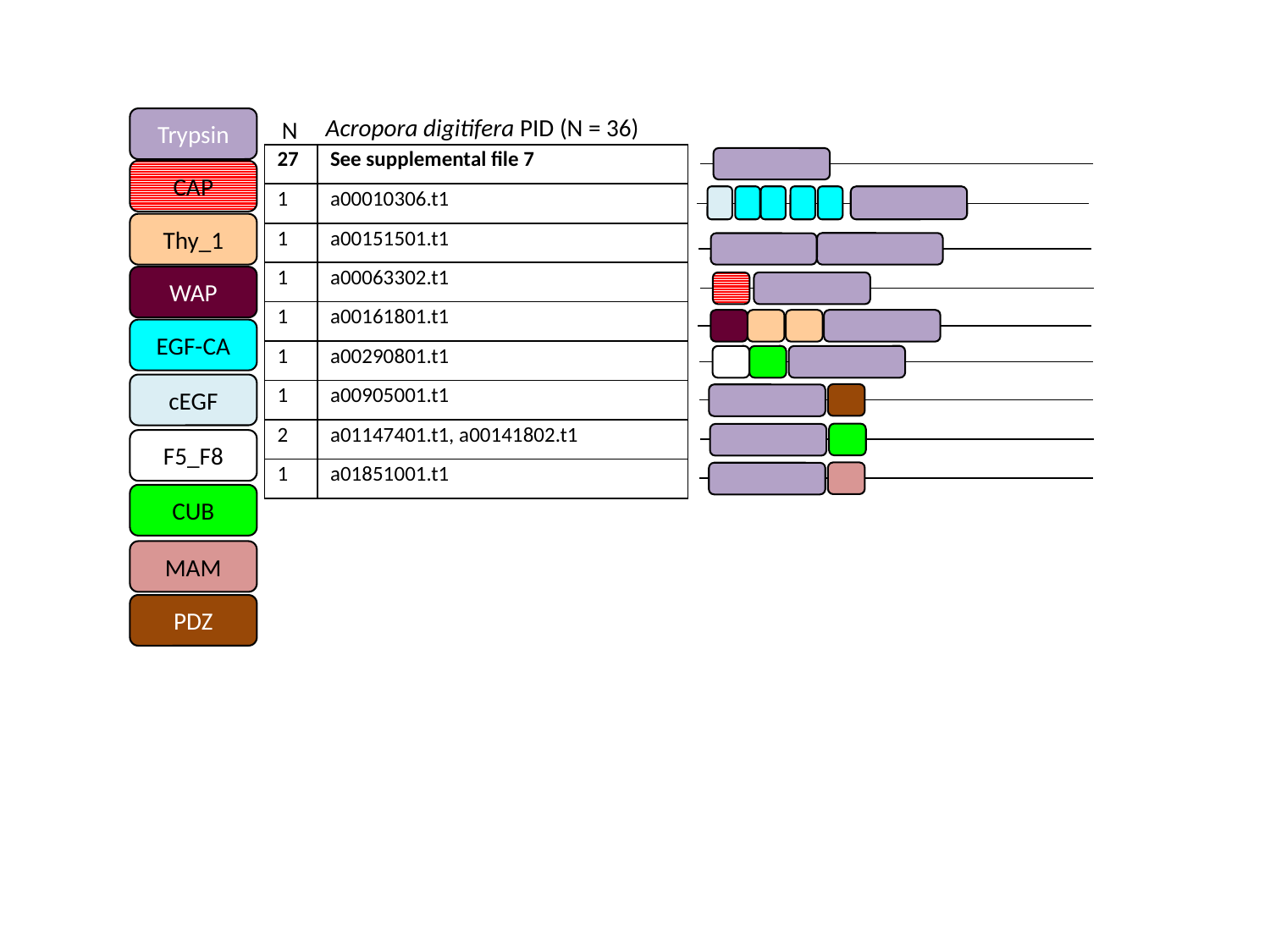

m.21993
Acropora digitifera PID (N = 36)
Trypsin
N
| 27 | See supplemental file 7 |
| --- | --- |
| 1 | a00010306.t1 |
| 1 | a00151501.t1 |
| 1 | a00063302.t1 |
| 1 | a00161801.t1 |
| 1 | a00290801.t1 |
| 1 | a00905001.t1 |
| 2 | a01147401.t1, a00141802.t1 |
| 1 | a01851001.t1 |
CAP
Thy_1
WAP
EGF-CA
cEGF
F5_F8
CUB
MAM
PDZ

## Slide 6
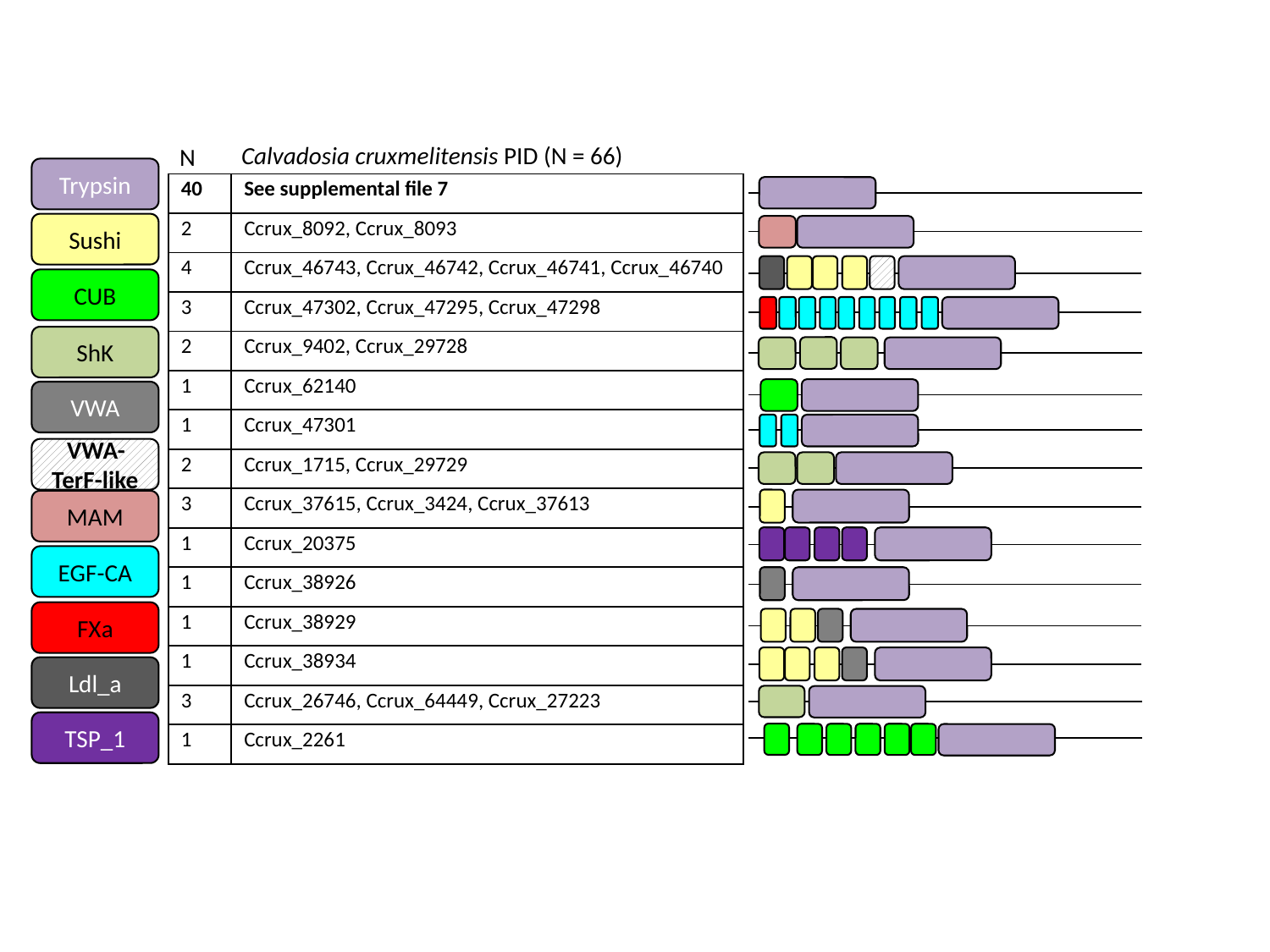

m.21993
Calvadosia cruxmelitensis PID (N = 66)
N
Trypsin
| 40 | See supplemental file 7 |
| --- | --- |
| 2 | Ccrux\_8092, Ccrux\_8093 |
| 4 | Ccrux\_46743, Ccrux\_46742, Ccrux\_46741, Ccrux\_46740 |
| 3 | Ccrux\_47302, Ccrux\_47295, Ccrux\_47298 |
| 2 | Ccrux\_9402, Ccrux\_29728 |
| 1 | Ccrux\_62140 |
| 1 | Ccrux\_47301 |
| 2 | Ccrux\_1715, Ccrux\_29729 |
| 3 | Ccrux\_37615, Ccrux\_3424, Ccrux\_37613 |
| 1 | Ccrux\_20375 |
| 1 | Ccrux\_38926 |
| 1 | Ccrux\_38929 |
| 1 | Ccrux\_38934 |
| 3 | Ccrux\_26746, Ccrux\_64449, Ccrux\_27223 |
| 1 | Ccrux\_2261 |
Sushi
CUB
ShK
VWA
VWA-TerF-like
MAM
EGF-CA
FXa
Ldl_a
TSP_1

## Slide 7
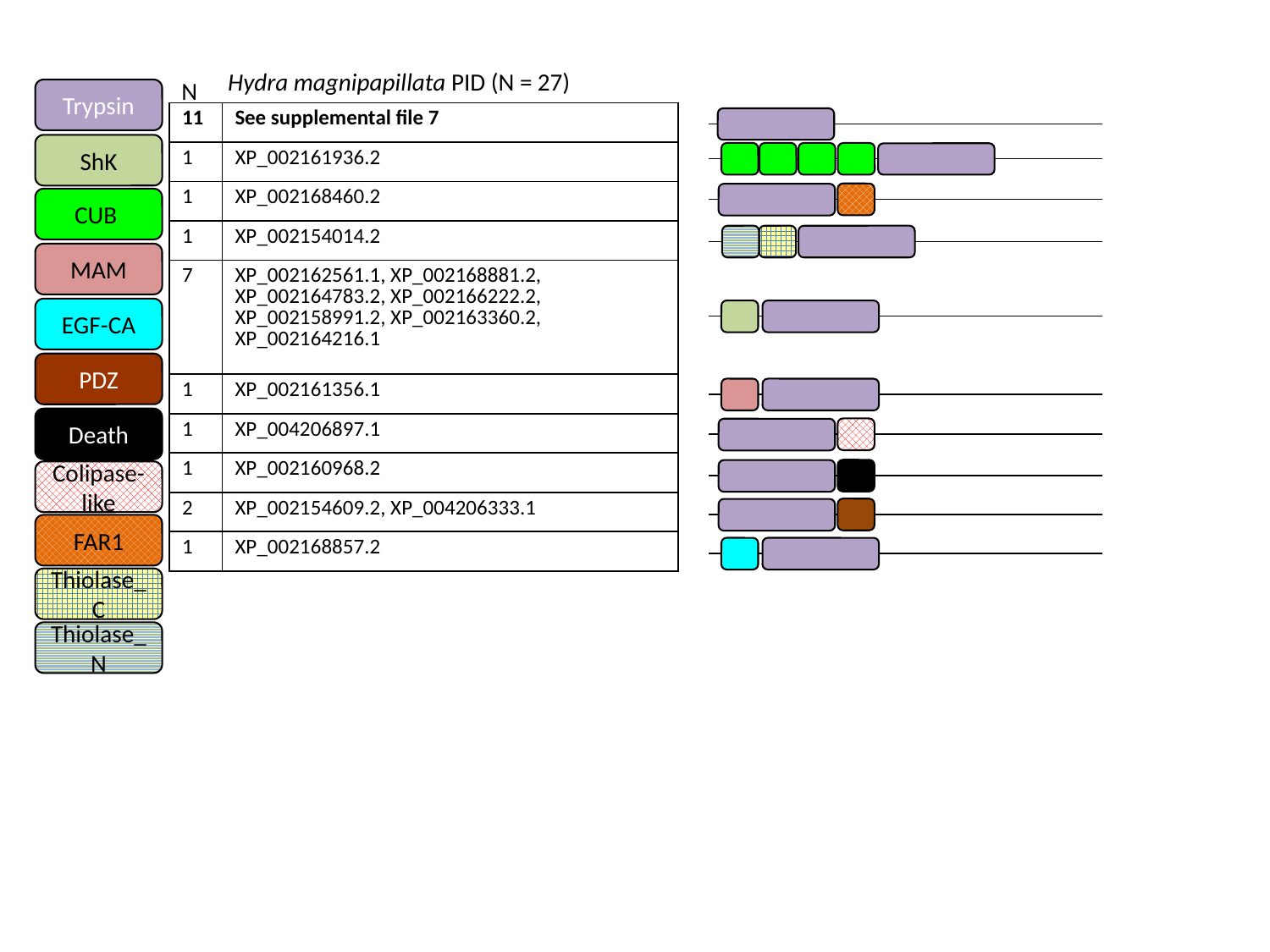

Hydra magnipapillata PID (N = 27)
N
Trypsin
| 11 | See supplemental file 7 |
| --- | --- |
| 1 | XP\_002161936.2 |
| 1 | XP\_002168460.2 |
| 1 | XP\_002154014.2 |
| 7 | XP\_002162561.1, XP\_002168881.2, XP\_002164783.2, XP\_002166222.2, XP\_002158991.2, XP\_002163360.2, XP\_002164216.1 |
| 1 | XP\_002161356.1 |
| 1 | XP\_004206897.1 |
| 1 | XP\_002160968.2 |
| 2 | XP\_002154609.2, XP\_004206333.1 |
| 1 | XP\_002168857.2 |
ShK
CUB
MAM
EGF-CA
PDZ
Death
Colipase-like
FAR1
Thiolase_C
Thiolase_N

## Slide 8
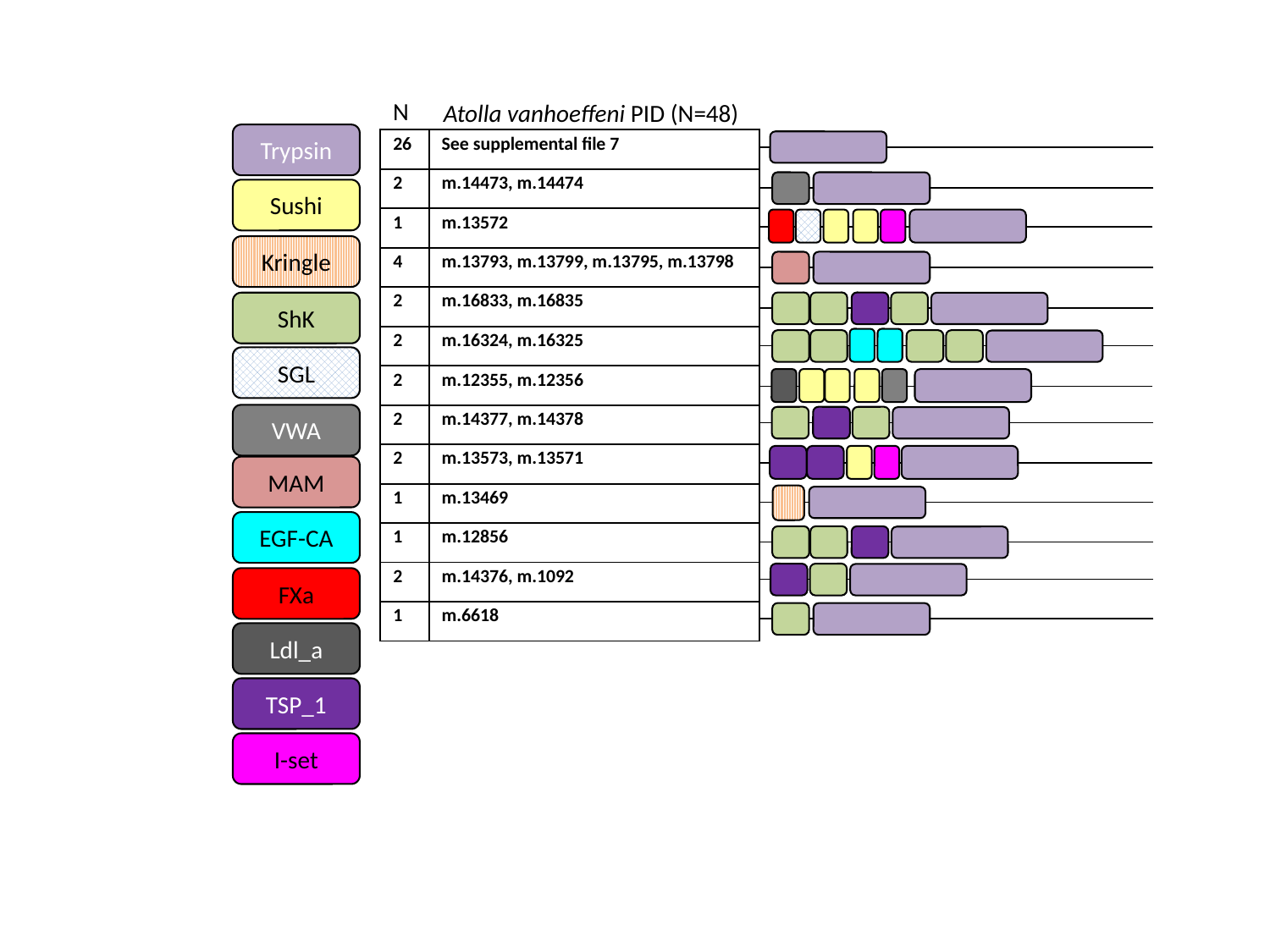

N
Atolla vanhoeffeni PID (N=48)
Trypsin
| 26 | See supplemental file 7 |
| --- | --- |
| 2 | m.14473, m.14474 |
| 1 | m.13572 |
| 4 | m.13793, m.13799, m.13795, m.13798 |
| 2 | m.16833, m.16835 |
| 2 | m.16324, m.16325 |
| 2 | m.12355, m.12356 |
| 2 | m.14377, m.14378 |
| 2 | m.13573, m.13571 |
| 1 | m.13469 |
| 1 | m.12856 |
| 2 | m.14376, m.1092 |
| 1 | m.6618 |
Sushi
Kringle
ShK
SGL
VWA
MAM
EGF-CA
FXa
Ldl_a
TSP_1
I-set

## Slide 9
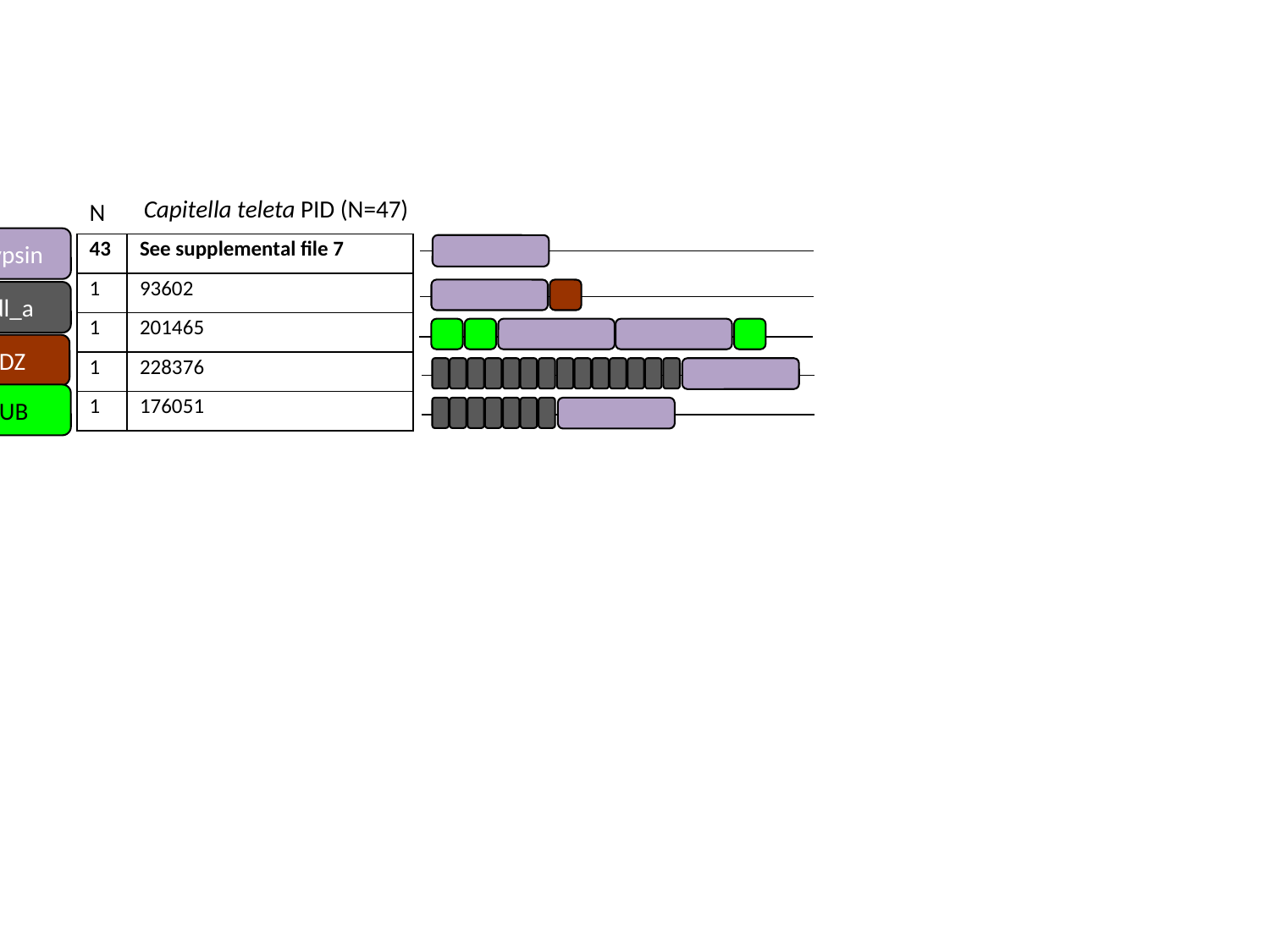

Capitella teleta PID (N=47)
N
Trypsin
Ldl_a
PDZ
CUB
| 43 | See supplemental file 7 |
| --- | --- |
| 1 | 93602 |
| 1 | 201465 |
| 1 | 228376 |
| 1 | 176051 |

## Slide 10
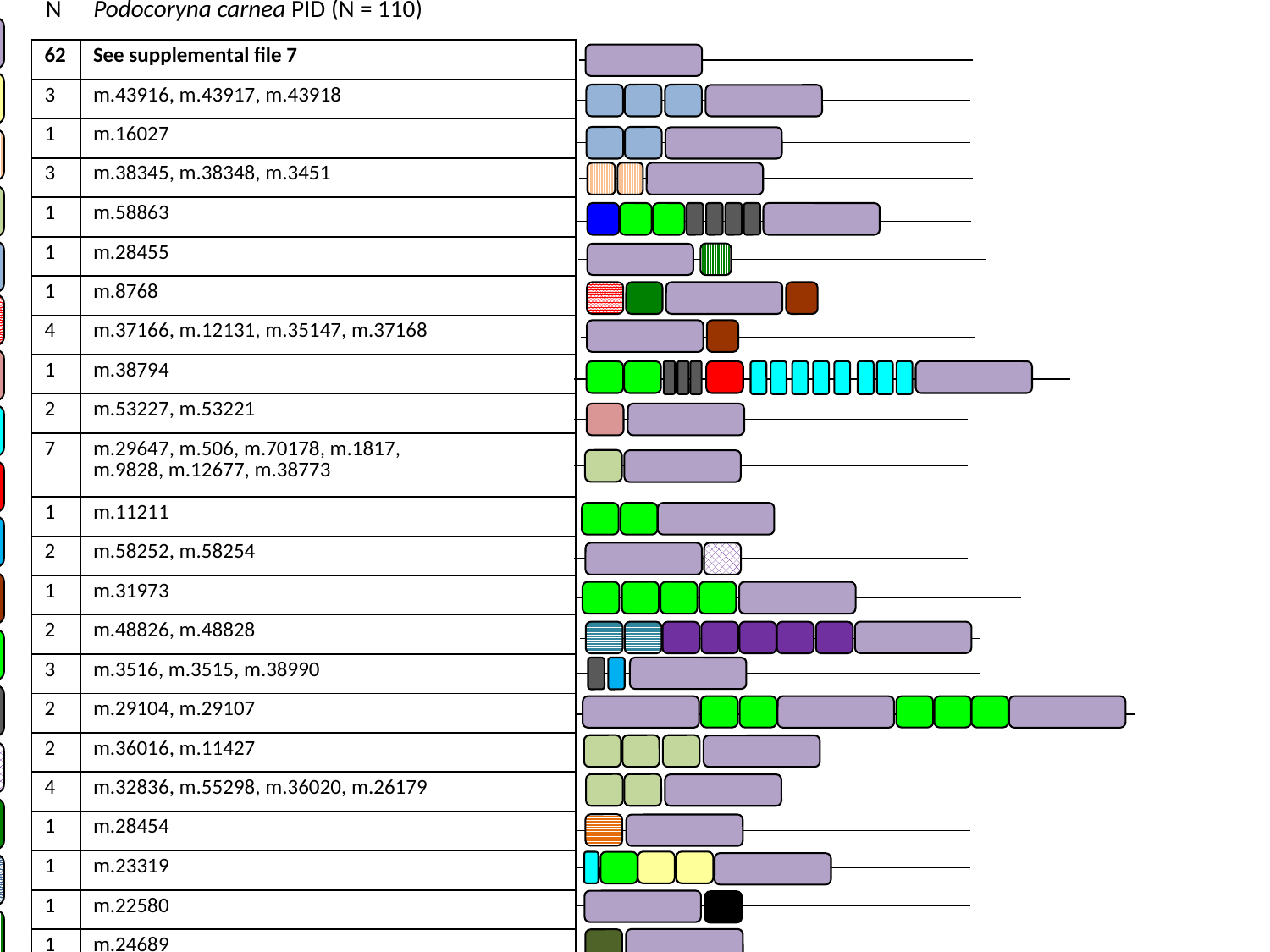

m.21993
N
Podocoryna carnea PID (N = 110)
Trypsin
| 62 | See supplemental file 7 |
| --- | --- |
| 3 | m.43916, m.43917, m.43918 |
| 1 | m.16027 |
| 3 | m.38345, m.38348, m.3451 |
| 1 | m.58863 |
| 1 | m.28455 |
| 1 | m.8768 |
| 4 | m.37166, m.12131, m.35147, m.37168 |
| 1 | m.38794 |
| 2 | m.53227, m.53221 |
| 7 | m.29647, m.506, m.70178, m.1817, m.9828, m.12677, m.38773 |
| 1 | m.11211 |
| 2 | m.58252, m.58254 |
| 1 | m.31973 |
| 2 | m.48826, m.48828 |
| 3 | m.3516, m.3515, m.38990 |
| 2 | m.29104, m.29107 |
| 2 | m.36016, m.11427 |
| 4 | m.32836, m.55298, m.36020, m.26179 |
| 1 | m.28454 |
| 1 | m.23319 |
| 1 | m.22580 |
| 1 | m.24689 |
| 1 | m.16474 |
| 1 | m.4072 |
| 1 | m.2429 |
Sushi
Kringle
ShK
Gal_lectin
IGFBP
MAM
EGF-CA
FXa
SRCR
PDZ
CUB
Ldl_a
Peptidase_S39
Kazal
EGF
Collagen
SMAP
LRR_5
TSP_1
Astacin
PLAT
SEA
Death
fn1
fn2

## Slide 11
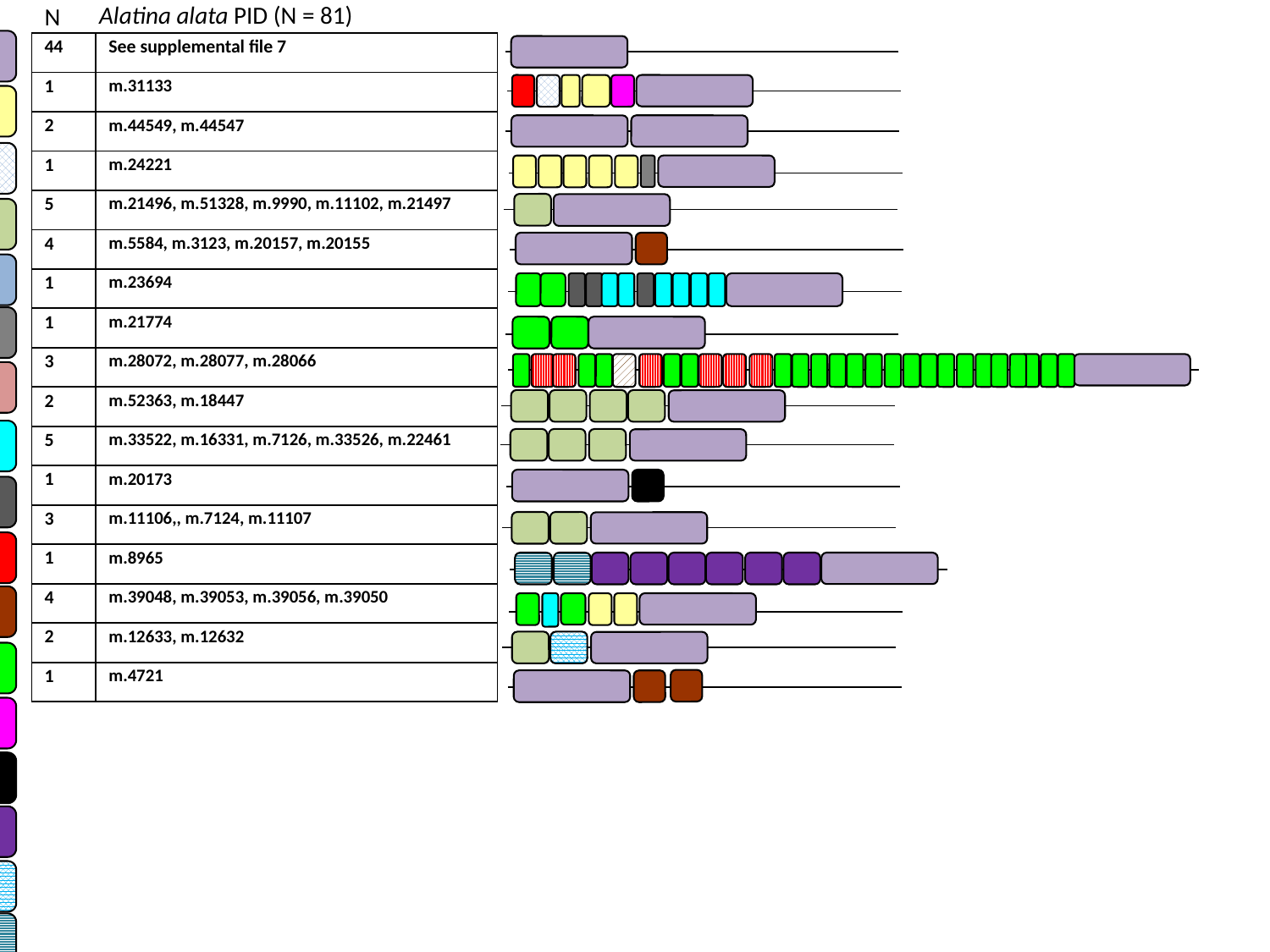

Alatina alata PID (N = 81)
N
Trypsin
| 44 | See supplemental file 7 |
| --- | --- |
| 1 | m.31133 |
| 2 | m.44549, m.44547 |
| 1 | m.24221 |
| 5 | m.21496, m.51328, m.9990, m.11102, m.21497 |
| 4 | m.5584, m.3123, m.20157, m.20155 |
| 1 | m.23694 |
| 1 | m.21774 |
| 3 | m.28072, m.28077, m.28066 |
| 2 | m.52363, m.18447 |
| 5 | m.33522, m.16331, m.7126, m.33526, m.22461 |
| 1 | m.20173 |
| 3 | m.11106,, m.7124, m.11107 |
| 1 | m.8965 |
| 4 | m.39048, m.39053, m.39056, m.39050 |
| 2 | m.12633, m.12632 |
| 1 | m.4721 |
Sushi
SGL
ShK
Gal_lectin
VWA
MAM
EGF-CA
Ldl_a
FXa
PDZ
CUB
I-set
Death
TSP_1
Med3
LRR_5
Hyd_WA
Tachylectin

## Slide 12
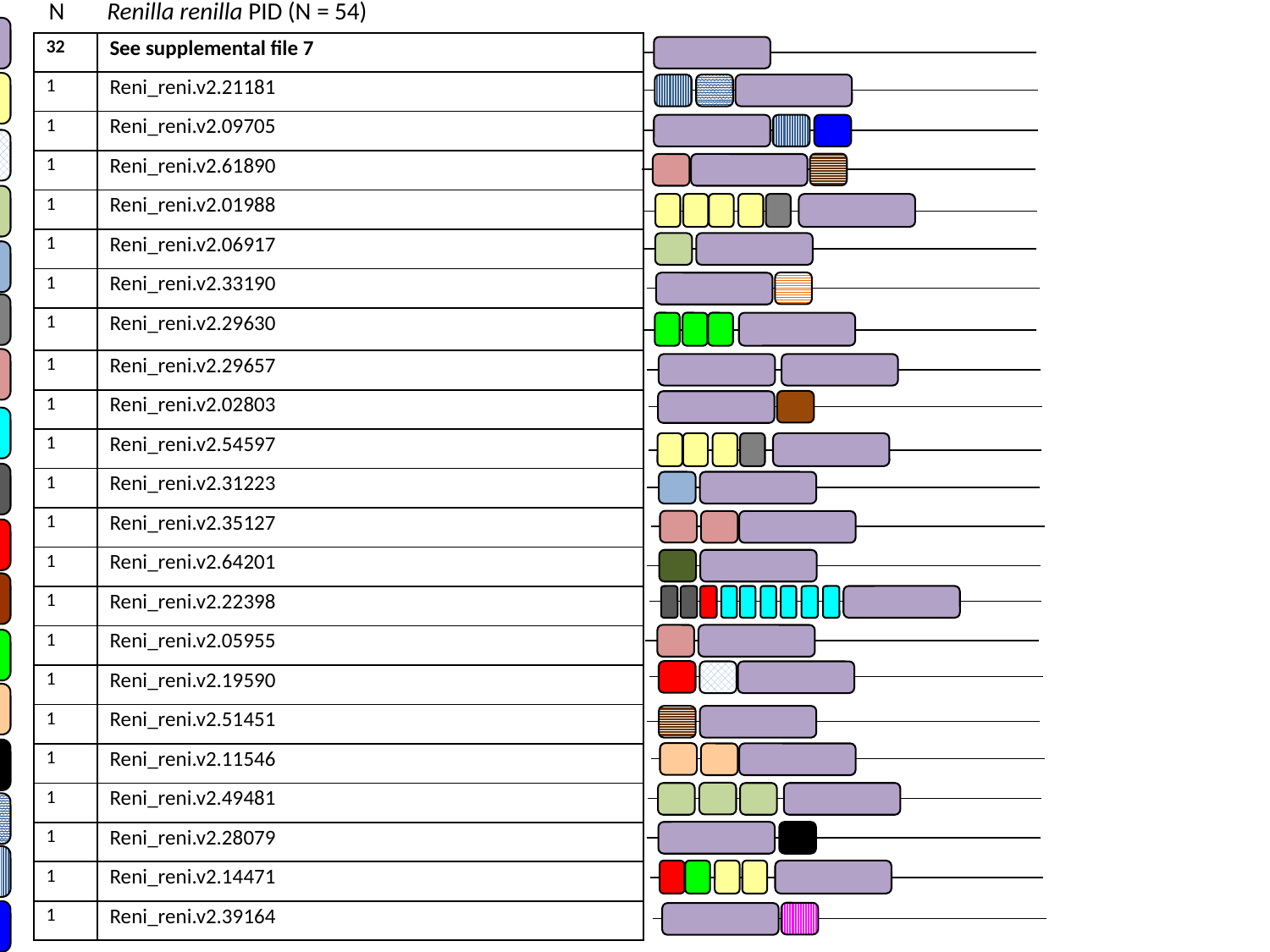

m.21993
N
Renilla renilla PID (N = 54)
Trypsin
| 32 | See supplemental file 7 |
| --- | --- |
| 1 | Reni\_reni.v2.21181 |
| 1 | Reni\_reni.v2.09705 |
| 1 | Reni\_reni.v2.61890 |
| 1 | Reni\_reni.v2.01988 |
| 1 | Reni\_reni.v2.06917 |
| 1 | Reni\_reni.v2.33190 |
| 1 | Reni\_reni.v2.29630 |
| 1 | Reni\_reni.v2.29657 |
| 1 | Reni\_reni.v2.02803 |
| 1 | Reni\_reni.v2.54597 |
| 1 | Reni\_reni.v2.31223 |
| 1 | Reni\_reni.v2.35127 |
| 1 | Reni\_reni.v2.64201 |
| 1 | Reni\_reni.v2.22398 |
| 1 | Reni\_reni.v2.05955 |
| 1 | Reni\_reni.v2.19590 |
| 1 | Reni\_reni.v2.51451 |
| 1 | Reni\_reni.v2.11546 |
| 1 | Reni\_reni.v2.49481 |
| 1 | Reni\_reni.v2.28079 |
| 1 | Reni\_reni.v2.14471 |
| 1 | Reni\_reni.v2.39164 |
Sushi
SGL
ShK
Gal_lectin
VWA
MAM
EGF-CA
Ldl_a
FXa
PDZ
CUB
Thy_1
Death
EGF
EGF_3
SEA
Glyco_hydro_65m
Ricin_B_
lectin
Herpes_gE
PLAT

## Slide 13
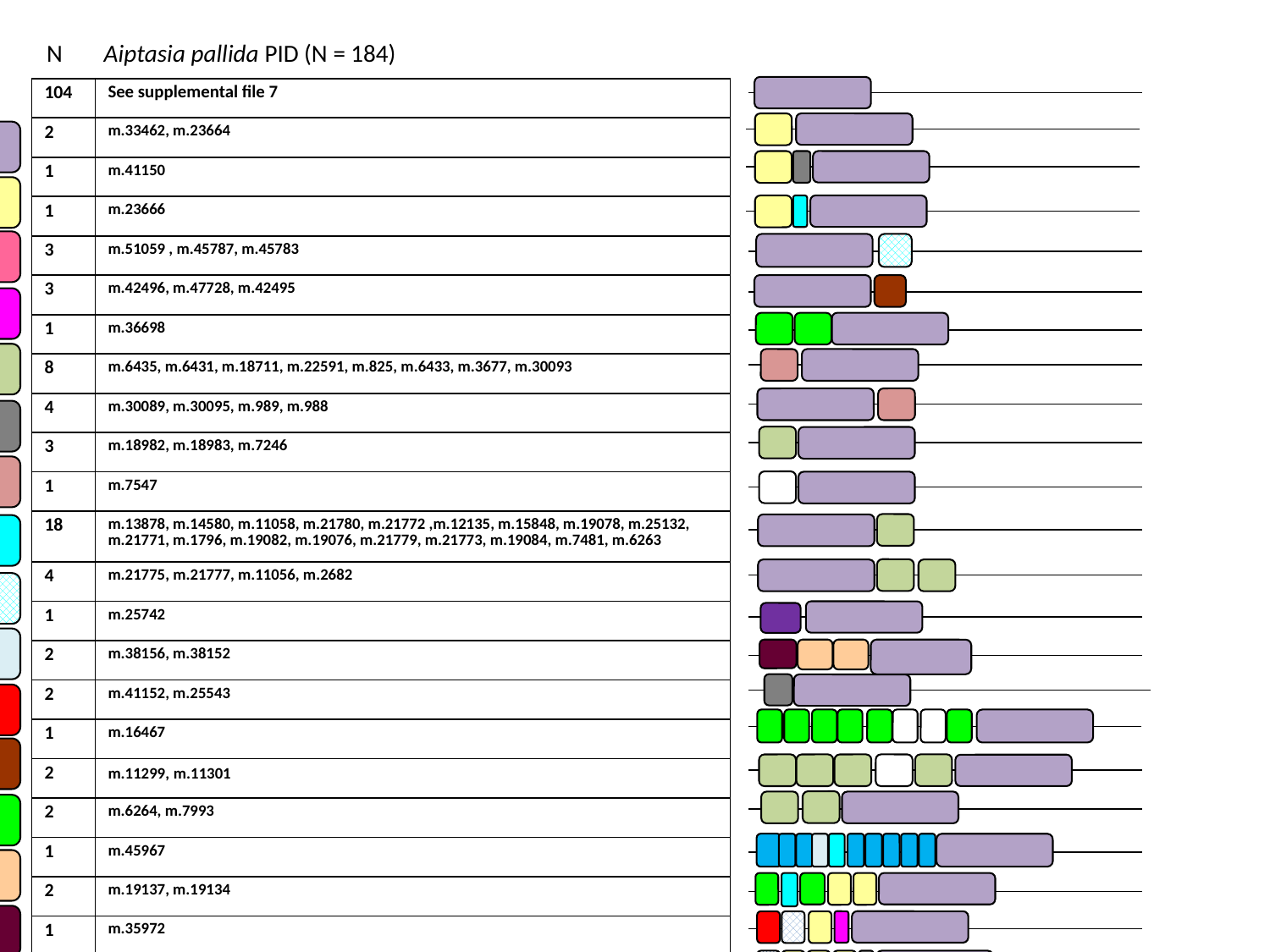

m.21993
N
Aiptasia pallida PID (N = 184)
| 104 | See supplemental file 7 |
| --- | --- |
| 2 | m.33462, m.23664 |
| 1 | m.41150 |
| 1 | m.23666 |
| 3 | m.51059 , m.45787, m.45783 |
| 3 | m.42496, m.47728, m.42495 |
| 1 | m.36698 |
| 8 | m.6435, m.6431, m.18711, m.22591, m.825, m.6433, m.3677, m.30093 |
| 4 | m.30089, m.30095, m.989, m.988 |
| 3 | m.18982, m.18983, m.7246 |
| 1 | m.7547 |
| 18 | m.13878, m.14580, m.11058, m.21780, m.21772 ,m.12135, m.15848, m.19078, m.25132, m.21771, m.1796, m.19082, m.19076, m.21779, m.21773, m.19084, m.7481, m.6263 |
| 4 | m.21775, m.21777, m.11056, m.2682 |
| 1 | m.25742 |
| 2 | m.38156, m.38152 |
| 2 | m.41152, m.25543 |
| 1 | m.16467 |
| 2 | m.11299, m.11301 |
| 2 | m.6264, m.7993 |
| 1 | m.45967 |
| 2 | m.19137, m.19134 |
| 1 | m.35972 |
| 1 | m.26197 |
| 1 | m.35973 |
| 2 | m.25497, m.25498 |
| 1 | m.44400 |
| 1 | m.28664 |
| 1 | m.25542 |
| 1 | m.26062 |
| 1 | m.33460 |
| 1 | m.29764 |
| 1 | m.14768 |
| 1 | m.12546 |
| 2 | m.25738, m.25740 |
| 1 | m.37592 |
| 1 | m.12721 |
| 1 | m.30094 |
Trypsin
Sushi
Ldl_b
I-set
ShK
VWA
MAM
EGF-CA
hEGF
cEGF
FXa
PDZ
CUB
Thy_1
WAP
SRCR
Ig_2
F5_F8
Astacin
EGF
TSP_1
Ldl_a
DUF 2360
Death
SGL

## Slide 14
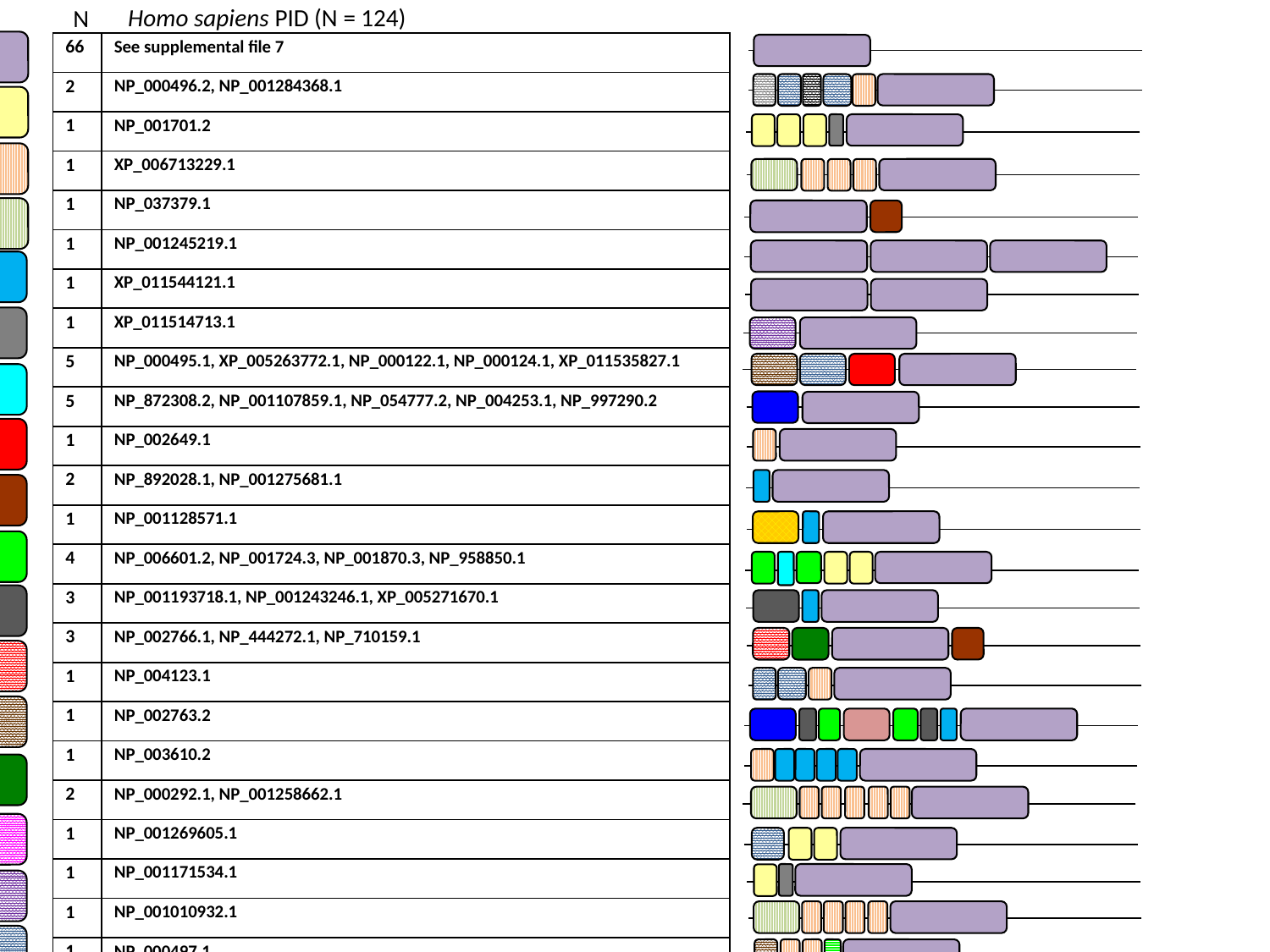

Homo sapiens PID (N = 124)
N
Trypsin
| 66 | See supplemental file 7 |
| --- | --- |
| 2 | NP\_000496.2, NP\_001284368.1 |
| 1 | NP\_001701.2 |
| 1 | XP\_006713229.1 |
| 1 | NP\_037379.1 |
| 1 | NP\_001245219.1 |
| 1 | XP\_011544121.1 |
| 1 | XP\_011514713.1 |
| 5 | NP\_000495.1, XP\_005263772.1, NP\_000122.1, NP\_000124.1, XP\_011535827.1 |
| 5 | NP\_872308.2, NP\_001107859.1, NP\_054777.2, NP\_004253.1, NP\_997290.2 |
| 1 | NP\_002649.1 |
| 2 | NP\_892028.1, NP\_001275681.1 |
| 1 | NP\_001128571.1 |
| 4 | NP\_006601.2, NP\_001724.3, NP\_001870.3, NP\_958850.1 |
| 3 | NP\_001193718.1, NP\_001243246.1, XP\_005271670.1 |
| 3 | NP\_002766.1, NP\_444272.1, NP\_710159.1 |
| 1 | NP\_004123.1 |
| 1 | NP\_002763.2 |
| 1 | NP\_003610.2 |
| 2 | NP\_000292.1, NP\_001258662.1 |
| 1 | NP\_001269605.1 |
| 1 | NP\_001171534.1 |
| 1 | NP\_001010932.1 |
| 1 | NP\_000497.1 |
| 1 | XP\_011518939.1 |
| 1 | NP\_705837.1 |
| 1 | NP\_127509.1 |
| 1 | NP\_005568.2 |
| 1 | NP\_001265514.1 |
| 2 | XP\_011530232.1, XP\_005262878.1 |
| 1 | NP\_068813.1 |
| 1 | XP\_011511056.1 |
| 1 | XP\_011526280.1 |
| 1 | XP\_011518365.1 |
| 1 | XP\_011530222.1 |
Sushi
Kringle
PAN_1
SRCR
VWA
EGF-CA
FXa
PDZ
CUB
Ldl_a
IGFBP
Gla
Kazal
Fz
V-set
EGF
MAM
SEA
GVQW
Thrombin_light
fn2
fn1
P12
